# *In vivo* programmed myeloid cells expressing novel chimeric antigen receptors show potent anti-tumor activity in preclinical solid tumor models

**DOI:** 10.3389/fimmu.2024.1501365

**Published:** 2024-12-13

**Authors:** Shannon Argueta, Yuxiao Wang, Hongyun Zhao, Neha Diwanji, Michael Gorgievski, Edward Cochran, Ewa Grudzien-Nogalska, Josephine D’Alessandro, Bruce McCreedy, Thomas Prod’homme, Robert Hofmeister, Jian Ding, Daniel Getts

**Affiliations:** Myeloid Therapeutics, Inc., Cambridge, MA, United States

**Keywords:** cancer therapy, lipid nanoparticles, myeloid cells, RNA therapeutics, cell therapy

## Abstract

**Introduction:**

The approval of chimeric antigen receptor (CAR) T cell therapies for the treatment of B cell malignancies has fueled the development of numerous *ex vivo* cell therapies. However, these cell therapies are complex and costly, and unlike in hematological malignancies, outcomes with most T cell therapies in solid tumors have been disappointing. Here, we present a novel approach to directly program myeloid cells *in vivo* by administering novel TROP2 CAR mRNA encapsulated in lipid nanoparticles (LNPs).

**Methods:**

The CAR comprises a TROP2 specific single-chain variable fragment (scFv) fused to a truncated CD89 which requires association with the FcRγ signal adapter to trigger myeloid-specific cell activation. The mRNA encoding the TROP2 CAR was encapsulated in LNPs. Co-immunoprecipitation, flow cytometry and enzyme-linked immunosorbent assay (ELISA) were used to measure CAR expression and functional activity *in vitro*. Anti-tumor efficacy of the TROP2 CAR mRNA/LNP was evaluated after intravenous administration in various murine tumor models.

**Results:**

*In vitro*, transient expression of the TROP2 CAR on monocytes triggers antigen-dependent cytotoxicity and cytokine release. In tumor bearing mice and cynomolgus monkeys, the TROP2 CAR mRNA/LNP are primarily expressed by myeloid cells. In a mouse xenograft model, intravenous administration of TROP2 CAR mRNA/LNP results in tumor growth inhibition and in a B16/F10-OVA immunocompetent melanoma mouse model, anti-tumor efficacy of a gp75-specific CAR correlates with increased number of activated T cells, activation of dendritic cells and a humoral response against B16/F10-OVA melanoma tumors.

**Discussions:**

These findings demonstrate that myeloid cells can be directly engineered *in vivo* to kill tumor cells and orchestrate an adaptive immune response and guide clinical studies for the treatment of solid tumors.

## Introduction

Chimeric antigen receptor (CAR) T cell therapies have provided tremendous benefits for patients with hematological malignancies underscoring the potential of T cells to reduce tumor growth and even cure cancers. The impressive results in liquid tumors spurred the development of many other T cell-focused approaches that tried to replicate the success of CAR-T cells in solid tumors. However, most of them failed to show clinical efficacy, either limited by upregulation of T cell-intrinsic inhibitory mechanisms or the highly immunosuppressive tumor microenvironment (TME). To overcome these barriers and complement T cell therapies, myeloid cells due to their ability to directly phagocytose tumor cells and coordinate an adaptive immune response, have gained in importance for cancer therapy.

Myeloid cells present a heterogeneous group of immune cells that comprises monocytes, macrophages, dendritic cells and granulocytes. These cells form part of the innate immune response and serve as the bridge to the adaptive immune response through their antigen presenting capabilities. In humans CD14^++^CD16^-^ classical monocytes, are the equivalent to inflammatory Ly6C^high^ monocytes in mice. Primarily originating in the bone marrow, they populate the peripheral blood. Attracted by chemokines, such as CCL2, they are rapidly recruited to sites of infection, injured tissue or tumors whereby Ly6C^high^ monocytes rapidly differentiate into dendritic cells, macrophages and other myeloid effector cells (1–4). In cancer, depending on local cues, infiltrating monocytes can differentiate into myeloid cells that can either inhibit or promote tumor growth. When appropriately stimulated, myeloid cells can differentiate into pro-inflammatory cells that have the potential to kill tumor cells via the production of cytokines such as TNF, phagocytosis or participation in antibody-dependent cytotoxicity. The secretion of inflammatory cytokines and presentation of antigens further orchestrates innate and adaptive cellular responses. However, under conditions of most established tumors, monocytes turn into tumor associated macrophages (TAM) and myeloid-derived suppressor cells (MDSCs) that promote cancer cell survival and suppress the establishment of an adaptive immune response (2, 5).

To exploit the tumoricidal potential of macrophages, in early studies monocyte-derived autologous macrophages were adoptively transferred to patients with solid tumors. Despite their cytotoxic activity *in vitro*, no significant anti-tumor responses were observed in patients (6–9). Hypothesizing that macrophages require additional factors to leverage their anti-tumor efficacy, with the advent of viral vector technologies macrophages were manipulated to express inflammatory factors, such as IFNα or IL-12 (10, 11). These engineered macrophages inhibited tumor growth in preclinical models but have not been tested in the clinic so far. Of note, due to challenges to transduce differentiated macrophages with lentiviral vectors (12), most therapeutic approaches used hematopoietic stem cells as starting material for macrophage engineering (13). Only more recently, human *in vitro* differentiated macrophages were successfully transduced with first-generation CD19 and HER2 CD3ζ CARs, respectively, using an adenoviral vector. Encouraged by positive preclinical data that demonstrated CAR-dependent phagocytosis, adoption of an inflammatory phenotype and a decrease in tumor burden in mouse xenograft models, the HER2-targeting CAR macrophages were advanced to clinical trials (14). In a combination study with pembrolizumab, the treatment had a favorable safety profile, but of the three patients presented, only one showed stable disease. The other two had progressive disease. Enrollment in the study has since been discontinued.

Compared to monocytes, the capacity of differentiated macrophages to infiltrate tumors is reduced. In addition, the number of manufactured cells in most cases is only sufficient for a single dose. Therefore, the focus has shifted to cell therapies starting with peripheral blood monocytes. CAR mRNAs delivered by lipid nanoparticles (LNPs) provide an attractive alternative to viral vectors. mRNA/LNP are readily taken up by monocytes and have been clinically validated in applications ranging from COVID-19 and therapeutic cancer vaccines to siRNA drugs (15–18). LNP-encapsulated RNA can be manufactured at lower costs than viral vectors and in quantities that support repeat dosing of patients. Finally, mRNA/LNP can be applied directly to patients, to program cells in the patient obviating any need for ex-vivo cell manipulation.

Here, we report preclinical data for a novel approach that utilizes myeloid cells to kill tumors and orchestrate an adaptive immune response. This is achieved through the *in vivo* delivery of a novel CD89 myeloid CAR. CD89 is a pleiotropic cell surface receptor for IgA, C reactive protein as well as pathogens including *Streptococcus pneumoniae* and *Escherichia coli*. Functionally, CD89 associates with the FcR γ chain, which contains an Immune Receptor Tyrosine Activation Motif (ITAM) signaling domain. During bacterial infection CD89 can serve as a mechanism to recognize and kill bacteria (19). In addition, dendritic cells can also express CD89, which mediates internalization and activation upon cross linking by IgA, presumably enhancing cross presentation of antigens (20). Notably, IgA made by tumor associated B cells can redirect myeloid cells against tumors leading to tumor cell death and enhanced anti-tumor immunity (21). The potent anti-tumor potential of CD89 activation is also being harnessed in novel bispecific antibodies. In preclinical studies, authors suggest that CD89 bispecific antibodies result in immediate innate immune activation and long-term adaptive engagement, together overcoming resistance mechanisms like immune checkpoint blockade and providing a robust therapeutic response against cancer (22, 23). To further leverage the potential of CD89 and the natural accumulation of myeloid cells in tumors, we engineered a novel myeloid specific CAR using CD89. Activating innate immunity while bridging adaptive immune responses could harness the dual power of immediate innate activation and long-term adaptive engagement.

Several design elements contribute to myeloid-specific programming and safety using non-targeted LNPs: (i) The CAR is delivered systemically using an mRNA/LNP formulation that is preferentially taken up by myeloid cells. (ii) A CD89-based CAR that only binds to tumor antigen, and to be functional requires the recruitment of the FcRγ signaling adapter which is endogenously expressed only in myeloid and NK cells. (iii) The mRNA/LNP can be dosed repeatedly for multiple rounds of myeloid cell programming. To test the therapeutic potential of CAR-programmed myeloid cells, we created a CAR against trophoblast cell surface antigen 2 (TROP2). TROP2 is a clinically validated target that has been successfully targeted with sacituzumab govitecan-hziy (Trodelvy^®^), a TROP2-directed antibody drug conjugate (ADC) approved for the treatment of metastatic triple-negative breast cancer and metastatic urothelial cancer (24). Monocytes expressing a TROP2 CAR were efficacious in xenograft models of human breast cancer. Furthermore, in a syngeneic mouse myeloma model, *in vivo* programmed anti-gp75 monocytes inhibited the growth of B16/F10-OVA melanoma tumors and created an inflammatory TME that enhanced the function of cytotoxic T cells and antigen presentation capacity of dendritic cells.

## Materials and methods

### Primary cells and cell lines

293T, Huh7 and HCC-1954 cells were purchased from American Type Culture Collection (ATCC) and were maintained in DMEM (Gibco) supplemented with 10% heat-inactivated Fetal Bovine Serum (HI-FBS, Gibco). SKOV3 cells were purchased from ATCC and were maintained in McCoy’s 5A Media (Gibco) supplemented with 10% HI-FBS (Gibco). THP-1 cells were purchased from ATCC and were maintained in suspension in Roswell Park Memorial Institute (RPMI)-1640 medium (Gibco) supplemented with 10% HI-FBS and 1% penicillin–streptomycin–L-glutamine (PSG) (Gibco). Reporter cell line THP1-Dual^®^ Cells and Jurkat-Dual™ Cells were purchased from InvivoGen. THP1-Dual^®^ Cells and Jurkat-Dual™ Cells were maintained in RPMI 1640 medium (THP1-Dual) or IMDM medium (Jurkat-Dual) supplemented with 10% FBS, 1% PSG, 25 mM HEPES and 100 μg/mL Normocin. Selection pressure for inserted reporter genes was maintained by adding 10 μg/mL of Blasticidin and 100 mg/mL of Zeocin antibiotics to the cells every other passage. The B16/F10-OVA tumor cell line was kindly provided by Dr. Michael Dee Gunn (Duke University Medical Center, Durham, NC). B16/F10-OVA tumor cells were maintained in DMEM supplemented with 10% FBS. All cells were maintained in an incubator at 37°C and 5% CO_2_ for the duration of culture.

Murine Ly6C^high^ bone marrow derived monocytes (BMDMs) were isolated from bone marrow. After flushing bone marrow cells from femurs and tibias of C57BL/6 mice (Charles River), the cells were first filtered through a 70 µm nylon cell strainer, and then monocytes were enriched using the EasySep™ Mouse Monocyte Isolation Kit (STEMCELL TECHNOLOGIES) according to the manufacturer’s instructions.

Whole blood from healthy human donors, collected into sodium heparin tubes, was purchased from Stemcell Technologies. Primary human PBMCs were isolated from donor Leukopaks using a Ficoll density gradient and aliquots were stored frozen in liquid nitrogen. Primary human monocytes were isolated by elutriation from donor Leukopak. Purity of isolated monocytes was confirmed by flow cytometry and cell aliquots were frozen in liquid nitrogen at -170°C. Upon thawing, PBMCs or monocytes were resuspended in TexMACS media (Miltenyi Biotec) containing 100 ng/mL human M-CSF (Miltenyi Biotec) and 2% Heat Inactivated Human AB serum (Gemini Bio) and allowed to recover for 16-20 hours at 37°C before electroporation.

### Antibodies and reagents

The primary antibodies used in co-immunoprecipitation were purchased as listed below: anti-FcεRI γ subunit rabbit polyclonal antibody (Upstate, Sigma), anti-FLAG mouse antibody (Sigma, clone M2) and anti-GAPDH rabbit polyclonal antibody (Cell Signaling). IR800 donkey anti-rabbit antibody and IR680 goat anti-mouse antibody were purchased from LiCor and used as secondary antibody in Western blot.

Antibodies used in biodistribution study with tumor free mice were mainly purchased from BioLegend, including anti-mouse CD3 PE-Cy5 (145-2C11), anti-mouse CD11c PE-Cy7 (N418), anti-mouse CD45.2 BV421 (104), anti-mouse CD45R/B220 APC/Fire750 (RA3-6B2), anti-mouse Ly6C AF700 (HK1.4), anti-mouse NK1.1 BV570 (PK136). Anti-mouse CD11b AF532 (M1/70) was purchased from Invitrogen. Anti-mouse CD80 BV480 (B7-1) was purchased from BD Biosciences. Anti-mouse Ly6G PerCP-Vio700 (REA526) was purchased from Miltenyi Biotech.

Antibodies used to detect CAR expression in tumor bearing NCG mice were purchased from BioLegend, including anti-mouse CD11c BV570 (N418), anti-mouse CD45 BV421 (30-F11), anti-mouse CD86 BV785 (GL-1), anti-mouse Ly6C AF700 (HK1.4), and anti-mouse Ly6G BV510 (1A8). Anti-mouse CD11b AF532 (M1/70) was purchased from Invitrogen.

Antibodies used to detect CAR expression in B16 tumor bearing mice as well as TME changes were mainly purchased from BioLegend, including anti-mouse CD3 PE-Cy5 (145-2C11), anti-mouse CD4 FITC (RM4-5), anti-mouse CD8 BV785 (53-6.7), anti-mouse CD11c PE-Cy7 (N418), anti-mouse CD24 PE-Cy5 (M1/69), anti-mouse CD40 APC/Fire 750 (3/23), anti-mouse CD45 BV421 (30-F11), anti-mouse CD86 BV785 (GL-1), anti-mouse TIM3 BV421 (RMT3-23), and anti-mouse TOX BV711 (TXRX10). Anti-mouse CD11b AF532 (M1/70), anti-mouse CD64 AF532 (X54-5/7.1), anti-mouse Granzyme B PE/Dazzle594 (QA16A02), and anti-Ki-67 PE-Cy7 (SolA15) were purchased from Invitrogen. Expression of gp75 CAR was detected with recombinant human gp75 (Sino Biological) conjugated with AF647 at Myeloid Therapeutics.

Antibodies used to detect CAR expression in NHP blood were mainly purchased from BD Biosciences, including anti-human CD3 BV421 (SP34-2), anti-human CD14 BV605 (M5E2), anti-human CD16 PE/Cy7 (3G8). Anti-human CD66abce FITC (TET2) was purchased from Miltenyi Biotec. Anti- human CD20 BV711 (2H7) and anti-human CD11b PE (ICRF44) were obtained from BioLegend.

Antibodies used to detect CAR expression in transfected human whole blood were mainly purchased from BioLegend, including anti-human CD3 APC/Fire750 (SK-7), anti-human CD19 PerCP-Cy5.5 (HIB19), anti-human CD45 FITC (HI30), anti-human CD56 PE-Cy7 (5.1H11). Anti-human CD14 BV421 (MφP-9) was purchased from BD Biosciences.

Flow cytometry detection of TROP2 CAR was performed using recombinant human TROP2 (ACRObio) that was conjugated with AF647 at Myeloid Therapeutics.

### CAR constructs

The TROP2 CAR is comprised of human GM-CSF signal peptide, a hRS7 derived scFv (V_H_-(G_4_S)_3_-V_L_), flexible linker (SGGGAAAGS) and truncated human CD89 (without extracellular domains 1 and 2). The TROP2 CAR used for co-immunoprecipitation (IP) is comprised of the human GM-CSF signal peptide, hRS7 derived scFv (VH-(G_4_S)_3_-V_L_), flexible linker with FLAG tag (SGGGGAAADYKDDDDKGS) and truncated human CD89 (without extracellular domains 1 and 2). Gp75 CAR is comprised of the mouse IgG kappa light chain signal peptide, TA99 derived scFv (V_L_-(G_4_S)_3_-V_H_) (25), flexible linker with FLAG tag (SGGGDYKDDDDKGS) and truncated human CD89 (same as in TROP-2 CAR). gBlocks encoding CARs were synthesized at IDT. Standard molecular biology techniques were used to clone all CAR constructs into the plasmid pcDNA3 backbone. All cloning steps were validated by restriction enzyme digestion and sequencing.

### Generation of mRNA

ARCA-capped TROP-2 CAR mRNA was synthesized at Aldevron. Gp75 CAR-encoding plasmids were linearized with BbsI restriction enzyme (New England BioLabs Inc.) and used as template for *in vitro* transcription (IVT). RNAs were synthesized in 4 mL reaction mixtures using T7 polymerase and HiScribe T7 ARCA mRNA Kit (New England BioLabs Inc.) for 1 hour at 37°C. Reaction mixtures were treated with 400 μL DNase I (New England BioLabs Inc.) for additional 30 minutes at 37°C, and RNAs were purified with a RNeasy maxi kit (QIAGEN). Poly(A) (~ 400As) was added enzymatically in the reaction containing 0.5 μg/μL RNA, 1X *E. coli* PAP Reaction buffer, 1 mM ATP and 0.5 U/μL *E. coli* PolyA Polymerase (New England BioLabs Inc.). Following 40 minutes incubation at 37°C, crude mRNAs were purified using RNeasy maxi kit (QIAGEN). To remove dsRNA, mRNAs were further purified on Agilent Technologies Series 1200 HPLC using RNASep™ Semi-Prep RNA Purification Column, (21.2 mm x 100 mm) (ADS Biotec) (26) and further recovered from collected fractions by centrifugation using Amicon Ultra Centrifugal Filter, 30 kDA MWCO (Milipore Sigma). The mRNAs were quality-assessed (4200 TapeStation System, Agilent Technologies Inc.) and quantitated spectrophotometrically (Nanodrop One Spectrophotometer, Thermo Fisher Scientific).

### LNP formulation

CAR encoding mRNAs were formulated into LNPs composed of ionizable cationic lipid, a polyethylene glycol (PEG) lipid, helper lipid and cholesterol. mRNA encoding gp75 CAR was formulated at Acuitas Therapeutics. MT-302, mRNA encoding TROP-2 CAR formulated in LNPs, was prepared at Arranta Bio.

### Electroporation of mRNA

THP1-Dual^®^ cells, Jurkat-Dual™ cells, human donor-derived monocytes and human donor-derived PBMCs were transfected with CAR mRNA by electroporation. Briefly, cells in culture were harvested, washed once with MaxCyte electroporation buffer, and mixed with 100 µg/mL of CAR mRNA for THP1-Dual cells, 50 µg/mL of CAR mRNA for Jurkat-Dual cells, or 200 µg/mL of CAR mRNA for primary human monocytes and primary human PBMCs. For FcRγ co-transfection experiments in Jurkat-Dual cells, 50 µg/mL of FcRγ mRNA was mixed with CAR mRNA for a total RNA concentration of 100 µg/mL per sample. The cell-mRNA mixture was added to the processing assembly cassette and immediately electroporated using a cell type specific protocol recommended by the manufacturer. Sterile nuclease-free water was used as a mock control for electroporation. Post-electroporation, the cells were allowed to recover in the processing assembly cassette for 10 minutes at 37°C, then transferred to culture vessel containing pre-warmed media and incubated further at 37°C.

Murine BMDMs were electroporated with CAR mRNA using Lonza 4D nucleofector system (Lonza). Enriched murine BMDMs were resuspended in P3 solution (Lonza) and mixed with 30 μg of CAR mRNA per 10^7^ cells on ice in a 100 µL volume. The cell-mRNA mixture was added to a nucleovette and immediately electroporated using program CM137. Sterile nuclease-free water was used as mock control for electroporation. After electroporation, BMDMs were resuspended in RPMI-1640 containing 10% HI-FBS, 1% PSG, 10 mM HEPES, and 1 mM Sodium Pyruvate and allowed to recover for 2 hours at 37°C before use in functional assays.

### Lipofectamine transfection

Huh7 and 293T cells were transfected with CAR mRNA by Lipofectamine MessengerMAX reagent (Invitrogen) following manufacturer’s recommendation. Briefly, 100,000 cells were seeded in a 24-well TC treated plate and incubated at 37°C overnight before transfecting with 0.5 µg (Huh7) or 1 µg (293T) of CAR mRNA with 1.5 µL of MessengerMAX reagent in OptiMEM (Gibco). For FcRγ co-transfection samples, equal concentration of FcRγ mRNA (0.5 µg for Huh7 cells or 1 µg for 293T cells) was mixed in along with CAR mRNA prior to adding the lipofectamine reagent. Transfected cells were incubated at 37°C and harvested at indicated timepoints for determining CAR cell surface expression.

### Phagocytosis assays

B16/F10-OVA cells were labeled with pHrodo™ Red dye (400 ng/mL) (Invitrogen) according to the manufacturer’s instructions. Murine Ly-6C^+^ BMDMs electroporated with gp75 CAR mRNA were co-cultured with pHrodo™ Red dye-labeled B16/F10-OVA cells at 1:1 E:T ratio (100,000 cells total) for 16-18 hours. All samples were set up in triplicates. After incubation, cells were collected and resuspended in FACS buffer, treated with Fc Block solution (BD) and stained with CD11b (Clone M1/70, BD Pharmingen), and acquired on Cytek NL-3000 (Cytek). Phagocytic index was calculated as % of phagocytic monocytes (pHrodo™ Red^+^CD11b^+^)/% of total monocytes (Total CD11b^+^) x 100. Mock-electroporated monocytes were co-cultured with B16/F10-OVA cells as controls for baseline phagocytosis.

### *In vitro* tumor co-culture assay

TROP2 antigen positive SKOV3-Luciferase tumor cells were used as targets in killing assay by control (Mock-transfected) or TROP2 CAR transfected PBMCs at a 5:1 effector-to-target (E:T) ratio. Three hours post-electroporation, transfected PBMCs were added on top of the tumor cells plated in sterile white opaque 96-well plate. All samples were set-up in triplicates. The coculture was incubated at 37°C for 72 hours. After coculture, supernatants were collected and cytokines were analyzed using the ProcartaPlex™ Human Cytokine & Chemokine Panel 1A, 34-plex Panel on a Luminex 200 instrument. To measure tumor killing, SKOV3 cell luciferase levels were measured using Bright-Glo™ Luciferase assay system (Promega). SpectraMax^®^ i3 (Molecular Devices) plate reader was used to measure luminescence signal. Effector-only wells and target-only wells were included as technical controls. SKOV3 luciferase levels for the coculture samples were normalized to target only levels and % Killing was calculated as follows:


% SKOV3 killing = (1 – Normalized luminescence for PBMC+tumor) x 100

### THP1 and Jurkat dual cell assay

THP1-Dual™ Cells are reporter cells derived from human THP-1 monocyte cell line by integration of two inducible reporter genes, IRF dependent Lucia luciferase reporter gene and NF-κB dependent secreted embryonic alkaline phosphatase (SEAP) reporter gene. Jurkat-Dual™ Cells are reporter cells derived from human Jurkat T cell line by integration of IRF dependent SEAP reporter gene and NF-κB dependent Lucia luciferase reporter gene. Therefore, these reporter cells can be used to evaluate activation of NF-κB and IRF pathways. The reporter cells were electroporated with TROP2 CAR mRNA, then stimulated with plate-bound rhTROP2 protein (10 μg/mL) at 3 hours post-electroporation. After 24 hours of stimulation at 37°C, the supernatant was collected from the culture and analyzed for SEAP activity by Quanti-Blue™ kit (InvivoGen), and for luciferase activity by QUANTI-Luc™ 4 Lucia/Gaussia kit (InvivoGen). TLR4 agonist LPS (InvivoGen) was used as positive control to activate NF-κB and 2’3’-cGAMP (InvivoGen) was used as positive control to activate IRF pathway for the THP1-Dual™ Cells, while TNFα (Preprotech) was used as positive control to activate NF-κB pathway and IFN-β (Preprotech) was used as positive control to activate IRF pathway for the Jurkat-Dual™ Cells.

### Co-immunoprecipitation of FcRγ with TROP2 CAR

TROP-2 CAR mRNA electroporated THP-1 cells were lysed at 20 hours post-electroporation with freshly prepared IP protein lysis buffer (Invitrogen). The cell lysate was incubated with anti-FLAG magnetic beads (Sigma) for 16-18 hours at 4°C. After washing, the beads were eluted with FLAG peptide solution (vendor, concentration) 3 times. The elutes were collected and run on NuPage 4-12% Bis-Tris pre-cast gel, then transferred onto a nitrocellulose membrane (Invitrogen). After overnight blocking with Fluorescent Blocking Buffer (Invitrogen), the membrane was blotted with Ab cocktail including anti-FcϵRI γ subunit rabbit polyclonal antibody (Upstate, Sigma) (1:1000), anti-FLAG mouse mAb (1:4000) and anti-GAPDH rabbit polyclonal Abs (1:1000). After overnight incubation with the primary antibody cocktail at 4°C, the membrane was washed and incubated with secondary antibody cocktail of IR800 donkey anti-rabbit antibody (1:10000) and IR680 goat anti-mouse antibody (1:10000) for 1 hour at room temperature. After the incubation, the blot was washed and imaged with Odyssey FC system (LiCor).

### mRNA-LNP transfection of human whole blood

Human whole blood was incubated with MT-302 (at 125 µg/mL) or PBS control for 3 hours at 37°C in presence of 3 µg/mL ApoE (PeproTech). At the end of incubation, the blood samples were lysed for 15 minutes in RBC lysis buffer at room temperature, washed with PBS, and then resuspended in RPMI containing 10% HI-FBS and 1X Gluta-Max-1 and cultured for 3 hours at 37°C. Cells were harvested on ice in DPBS containing 2 mM EDTA, and were analyzed for CAR expression by flow cytometry.

### *In vivo* studies

All *in vivo* studies were carried out under accordance with approved IACUC protocols.

#### Biodistribution study of TROP2 CAR/LNP with non-tumor-bearing C57BL/6 mice

Six-to-eight weeks old female C57BL/6 mice (The Jackson Laboratory) were dosed with TROP2 CAR/LNP (at 0.5 or 1 mg/kg/dose) or PBS via intravenous (i.v.) infusion (100 μL per mouse) into the lateral tail vein. At 6 hours post infusion, blood, bone marrow and spleen were harvested from the animals. Single cell suspensions were obtained from all harvested organs. CAR expression by different immune cell populations were evaluated by flow cytometry.

#### Anti-tumor efficacy study of TROP2 CAR/LNP in human breast cancer xenograft model

Six-to-eight weeks old female NCG mice (Charles River Laboratories) were subcutaneously inoculated with 2 x 10^6^ of HCC-1954 tumor cells into the right dorsal flank of each mouse. Tumor measurements with vernier calipers were recorded three times weekly starting on day 17 post-implant. Tumor volumes were calculated for a hemi-ellipsoid, using the formula: Volume = (d^2^×D)/2. When mean tumor volume reached 50 -150 mm^3^, mice were randomized into treatment groups, and administered with PBS, empty LNP, or TROP2 CAR/LNP at 1 mg/kg/dose every 4 days (Q4D) or every 2 weeks (Q2W) via i.v. injection (100 μL per mouse) into the lateral tail vein.

To measure TROP2 CAR expression *in vivo*, NCG mice with established HCC-1954 tumors (tumor volume approximately 500 mm^3^) were dosed i.v. with TROP2 CAR/LNP (1 mg/kg) or PBS. Six hours post infusion, blood, spleen, bone marrow and tumor were harvested. Single cell suspensions were prepared from collected tissues and stained for CAR expression and analyzed by flow cytometry.

#### Anti-tumor efficacy study for gp75 CAR mRNA-LNP in mouse syngeneic melanoma model

Six-to-eight weeks old female C57BL/6 mice (The Jackson Laboratory) were subcutaneously inoculated with 2.5 x 10^5^ of B16/F10-OVA tumor cells into the right dorsal flank of each mouse. Tumor measurements with vernier calipers were recorded three times weekly starting day 17 post inoculation. Tumor volumes were calculated for a hemi-ellipsoid, using the formula: Volume = (d^2^×D)/2. When mean tumor volume reached 50 -100 mm^3^, mice were randomized into treatment groups and treated with PBS, Empty LNP, or gp75 CAR mRNA-LNP at 2 mg/kg every other day (Q2D) by i.v. injection. At 24 hours following the last injection, the animals were euthanized to collect the tumors and spleens. Single cell suspension was obtained from the tumors and analyzed by flow cytometry for T cell activation, exhaustion and proliferation. Spleen single cell suspensions were obtained and analyzed by flow cytometry for dendritic cell activation.

For anti-OVA IgG analysis, tumor bearing mice were administered with 1 mg/kg/dose gp75 CAR mRNA-LNP, 3XSTOP gp75 CAR mRNA/LNP or Empty LNP i.v. every 7 days. Blood was collected from treated animals at 48 hours post 2^nd^ injection. Production of anti-OVA IgG was measured using Anti-OVA IgG1 ELISA plates (Chondrex) per manufacturer’s instructions.

#### *In vivo* CAR expression in non-human primate (NHP) following TROP2 CAR/LNP infusion

Naïve cynomolgus monkeys were infused with PBS or TROP2 CAR/LNP (at 0.1, 0.5 or 1 mg/kg/dose) at Charles River Laboratory (Mattawan, MI). Blood samples were collected from animals at 12 hours post infusion. CAR expression was analyzed by flow cytometry for T cells, B cells, NK cells, granulocytes and monocytes in the blood.

### Flow cytometry

Flow cytometry was used to evaluate CAR expression *in vitro* for THP-1 cells, murine BMDMs, human primary monocytes, PBMCs or whole blood that were electroporated with mRNA or transfected with mRNA-LNP. Additionally, flow cytometry was also utilized to evaluate CAR expression *ex vivo* for tissues harvested from mice or cynomolgus monkeys after injection with TROP2 CAR/LNP. Harvested cells were stained with 7-AAD or Zombie NIR for viability, Fc blocked, then with Ab cocktails for lineage markers as well as for CAR. Recombinant human TROP2 (rhTROP2) protein conjugated with Alexa Fluor 647 was used to detect the TROP2 CAR. An anti-FLAG antibody was used to detect the Flag-tagged gp75 CAR.

## Results

### Development of anti-TROP2-CD89 CAR

**Figure 1:**
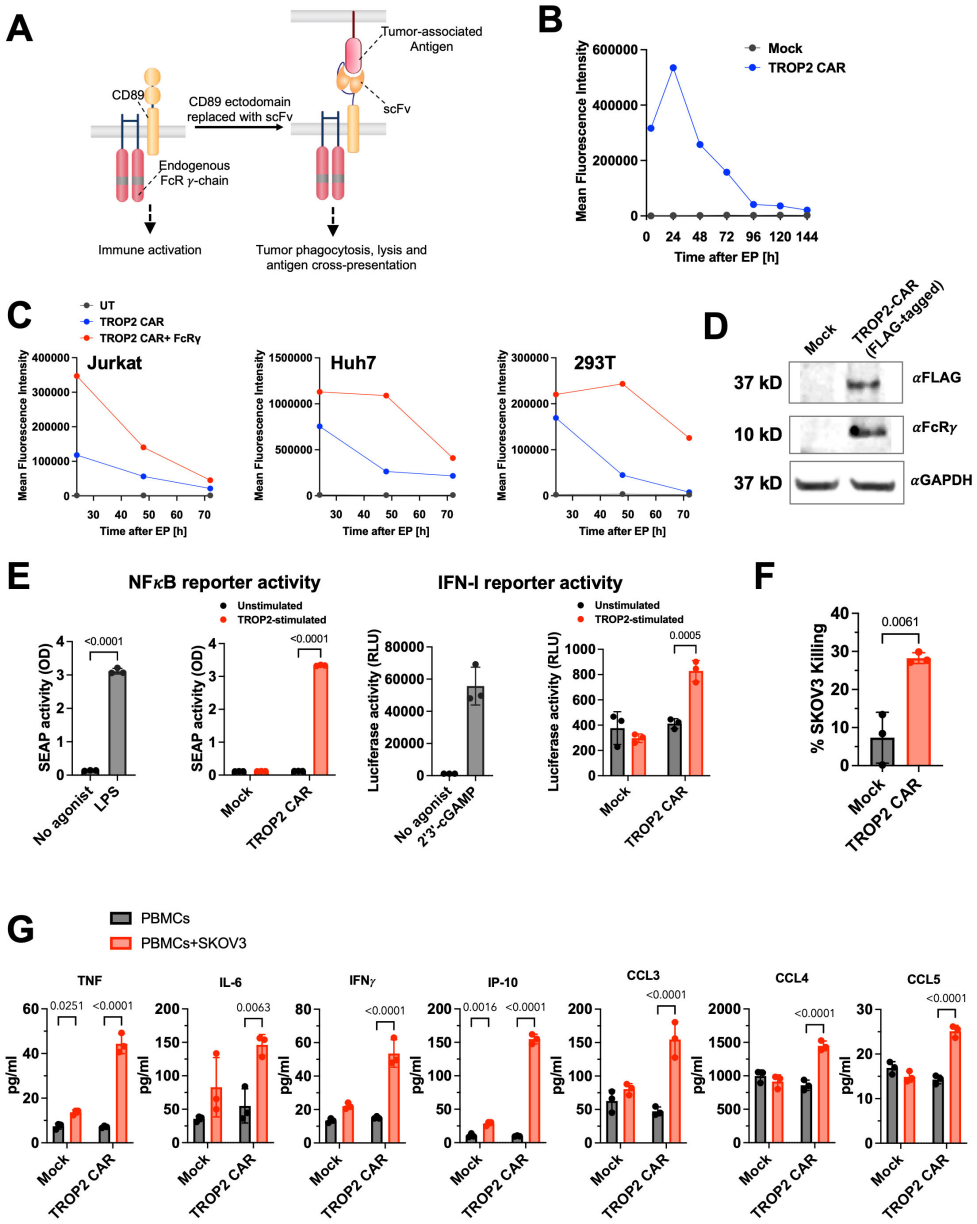
Development of CD89 (Fc*α*R) based CAR targeting TROP2. **(A)** Design of the CD89 fusion CAR. Human IgA receptor Fc*α*R (CD89) forms multi-chain complexes with endogenous FcR*γ*. CD89-based CAR is constructed by replacing the ectodomain of CD89 with a tumor-targeting scFv. **(B)** TROP2-CD89 CAR was delivered to primary human monocytes via mRNA electroporation and CAR expression was measured using flow cytometry. Data shown are representative of two different donors. Also see **Supplementary Figure 1A**. **(C)** Expression level and duration of TROP2 CAR are influenced by the presence of FcR*γ*. TROP2 CAR mRNA was transfected into Jurkat, Huh7 or 293T cells with or without co-transfection of FcR*γ* mRNA. Expression of TROP2 CAR was measured using flow cytometry over 3 days. **(D)** Association of TROP2 CAR with endogenous FcR*γ* detected by co-immunoprecipitation. Western Blot was performed against the FcR*γ* chain with *α*GAPDH as loading control. **(E)** TROP2 CAR induced NF-*κ*B and IFN-I pathways activation as measured in the THP1-Dual™ reporter cells. Stimulation with the TLR4 agonist LPS or cGAS agonist 2’3’-cGAMP are shown as positive control. Statistical analysis was performed by Two-Way ANOVA followed by Sidak’s multiple comparisons test for comparing unstimulated and TROP2-stimulated conditions, and an unpaired t-test (two-tailed) for comparing no agonist vs agonist stimulated conditions. **(F)** Co-culture of PBMCs electroporated with TROP2 CAR with SKOV3 tumor cells results in elevated tumor killing compared to mock electroporated PBMCs. Statistical analysis was performed using an unpaired t-test (two-tailed). **(G)** Co-culture of PBMCs electroporated with TROP2 CAR with SKOV3 tumor cells results in production of pro-inflammatory cytokines and chemokines. Statistical analysis was performed by Two-Way ANOVA followed by Sidak’s multiple comparisons test. All data shown are mean ± SD. Statistical comparison with p value <0.05 are labeled.

We investigated the therapeutic potential of *in vivo* programmed myeloid cells that express a trophoblast cell surface antigen 2 (TROP2) targeting CAR in solid tumor models. TROP2 is overexpressed in many human solid cancers (27) and has been clinically validated by antibody drug conjugate approaches (28). Different from earlier CD3ζ-based CARs used for engineering of myeloid cells (29), the CAR construct in the present study comprises a TROP2-specific single-chain variable fragment (scFv) tethered to an N-terminally truncated version of CD89 (FcαR) (**Figure 1A**). Importantly, CD89 was chosen as a bona fide immune cell receptor whose expression is restricted to myeloid cells and integrated into myeloid-specific signaling circuits. CD89 is functionally associated with the ITAM motif containing signaling adapter FcRγ to activate myeloid cells (30).

To evaluate the expression of the CAR mRNA, primary human monocytes were electroporated with TROP2 CAR mRNA. CAR expression levels peaked 24 hours after electroporation. While the expression level decreased over time, as expected with an mRNA encoded construct, robust expression on the cell surface was still observed seven days later. At that time, > 80% of the cells were CAR positive (**Figure 1B**; **Supplementary Figure 1A**). Based on earlier literature reports that describe the stabilization of Fc receptor expression by FcRγ (31), we investigated TROP2 CAR surface expression in cell types that lack the expression of the FcRγ adapter protein, including 293T, Huh7, and Jurkat cells. Consistent with these reports, we found transient TROP2-CD89 fusion CAR expression in cells without FcRγ, whereas co-transfection of FcRγ enhanced expression levels and duration of expression (**Figure 1C**).

Next, we tested whether the CD89-based CAR, like the natural CD89, associates with the FcRγ signal adapter molecule to trigger cell activation. To facilitate pull-down experiments using electroporated THP-1 cell lysates, the TROP2 CAR was Flag-tagged. As shown in the Western blot in **Figure 1D**, the anti-Flag antibody pulled down the TROP2 CAR and FcRγ only in cells that were electroporated with the TROP2 CAR mRNA, not in mock electroporated THP-1. Detection of the FcRγ chain at the expected size of 10 kDa confirmed the recruitment of the endogenous FcRγ adapter protein by the CAR in THP-1 cells (**Figure 1D**).

Downstream signaling of the TROP2 CAR through FcRγ chain was further evaluated in THP-1-Dual^®^ cells. THP1-Dual^®^ cells allow the simultaneous study of the NF-κB pathway, by monitoring the activity of secreted embryonic alkaline phosphatase (SEAP), and the IRF pathway, by assessing the activity of secreted Lucia luciferase. More than 90% of TROP2 CAR mRNA electroporated THP1-Dual™ cells expressed the antigen receptor (**Supplementary Figure 1B**). Robust activation of NF-κB was observed in CAR mRNA electroporated cells upon stimulation with plate-bound recombinant human TROP2 as indicated by SEAP activity in the cell culture supernatant (**Figure 1E**). The level of NF-κB induction was comparable to the level induced by the Toll-like receptor agonist LPS. Mock electroporated cells did not secrete SEAP. The absence of NF-κB reporter signal in unstimulated cells confirmed the antigen-dependent activation of cells. IRF reporter activity was also significantly increased, although to a much lower extent than the NFκB reporter gene (**Figure 1E**). The lack of NFκB signal activation in CAR-transfected Jurkat-Dual^®^ cells (**Supplementary Figure 1C**), a T cell reporter cell line which lacks FcRγ further confirms CAR signaling through FcRγ (**Supplementary Figure 1B**).

To investigate the expression profile and anti-tumor activities of the TROP2 CAR in blood cells, human PBMCs isolated from healthy donors were transfected with TROP2 CAR mRNA via electroporation. Flow cytometric analysis showed robust expression of the TROP2 CAR in CD14^+^ monocytes (**Supplementary Figure 1D**). Under these *in vitro* transfection conditions, we also observed low TROP2 CAR expression on NK, T cells or B cells (**Supplementary Figure 1D**). TROP2 CAR-transfected PBMCs lysed target positive SKOV3. (**Figure 1F**). Co-culture of transfected PBMCs with SKOV3 cells induced the production of inflammatory cytokines (TNF, IL-6, IFNγ, IP-10) and the chemokines CCL3, CCL4 and CCL5 indicative of an inflammatory response triggered by the CAR (**Figure 1G**). In the absence of target cells, transfected PBMCs produced baseline cytokines comparable to mock transfected cells. Collectively, we demonstrated that the novel anti-TROP2 CAR is functional *in vitro*.

### TROP2 CAR is preferentially expressed in myeloid cells *in vitro* and *in vivo*

**Figure 2:**
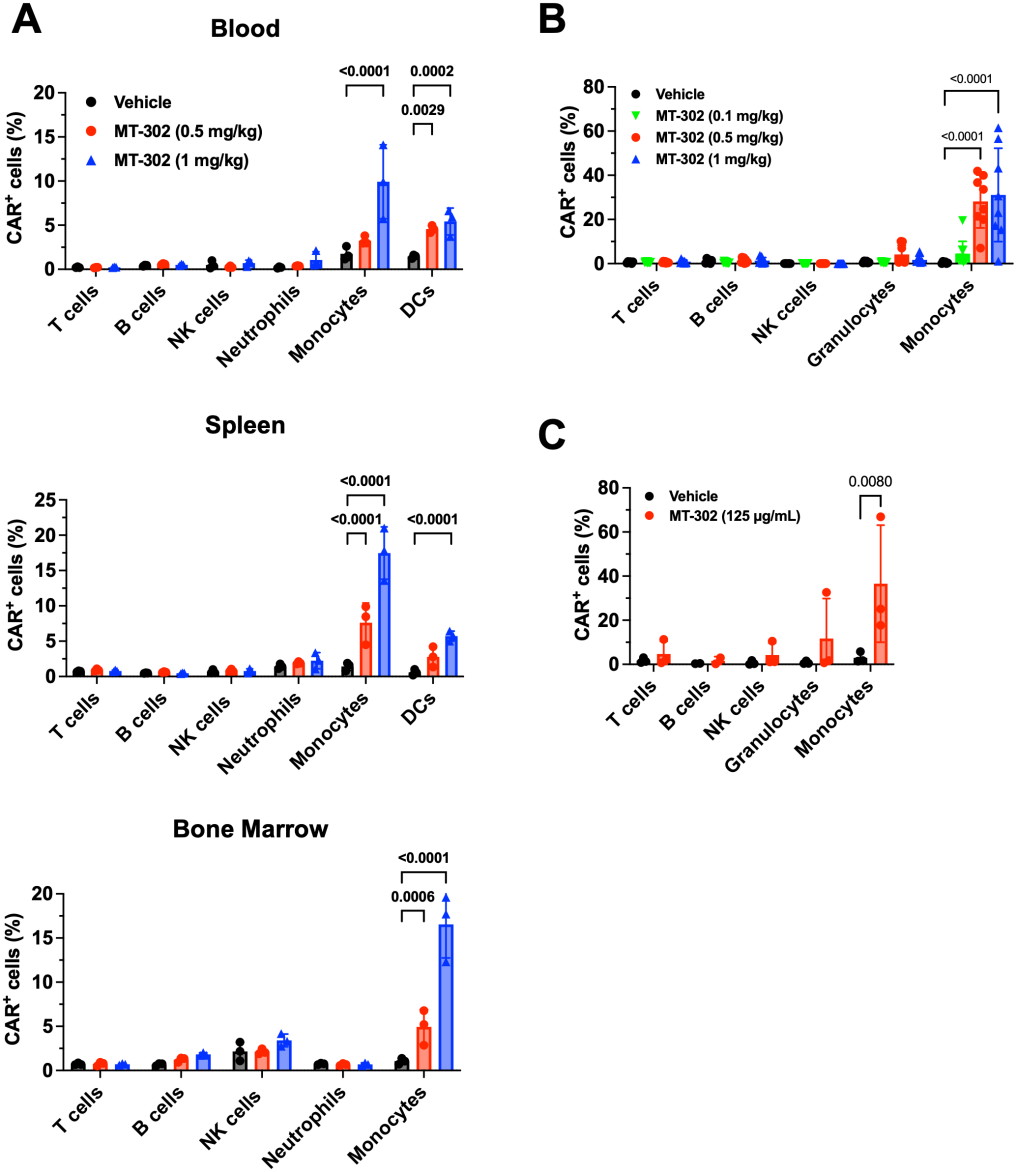
Myeloid cell-targeted delivery of TROP2 CAR mRNA/LNP. **(A)** *In vivo* CAR expression in immune competent mice resulted from MT-302 infusion. Non-tumor bearing C57BL/6 mice were given a single i.v. injection of MT-302 at 0.5 mg/kg or 1 mg/kg. Blood, spleen, bone marrow and liver were collected from treated mice at 6 hours post-infusion. Single cell suspensions were prepared and stained for CAR expression among immune subsets (T cells, B cells, NK cells, Neutrophils, Monocytes, DCs) by FACS. Data shown were average and STD of each group (n=3 mice per group). Statistical analysis was performed by Two-Way ANOVA followed by Sidak’s multiple comparisons test. **(B)** *In vivo* CAR expression in non-human primate resulted from MT-302 infusion. Naïve cynomolgus monkeys were infused with MT-302 (i.v.) (0.1, 0.5, or 1 mg/kg) for 1h. Blood was collected 12 hours after infusion and TROP2 CAR surface expression of different immune populations of PBMCs was analyzed by FACS. Data shown were average and STD of each group (10 monkeys, 5 females and 5 males). Statistical analysis was performed by Two-Way ANOVA followed by Dunnett’s multiple comparisons test. **(C)** CAR expression in human blood resulted from *in vitro* transfection. Whole blood from healthy human donors was incubated with 125 µg/mL of MT-302 for 3 hours in presence of ApoE3 (at 1.5 µg/mL). Following RBC lysis and additional 3 hours culture, surface TROP-2 CAR expression was assessed by FACS for T cells, B cell, NK cells, Granulocytes and Monocytes. Data shown were average and STD of each group (n=3 donors). Statistical analysis was performed by Two-Way ANOVA followed by Sidak’s multiple comparisons test.

Next, we explored the expression of TROP2 CAR in different cellular compartments in mice. TROP2 CAR mRNA/LNP were injected intravenously into non-tumor bearing C57BL/6 mice at 0.5 mg/kg and 1 mg/kg. Blood and tissues were harvested 24 hours later, and TROP2 CAR expression was determined by flow cytometry. Ly6C^+^ monocytes presented the largest CAR^+^ cell population in peripheral blood, spleen and bone marrow (**Figure 2A**). The mean percentage of TROP2 CAR expressing cells ranged between 10-15% at 1 mg/kg, and 5-7% at 0.5 mg/kg. CD11c^+^MHCII^hi^ dendritic cells (DCs) were the second largest cell population expressing the CAR in blood and spleen (**Figure 2A**). Other immune cell populations expressed very little to no CAR demonstrating that the TROP2 CAR mRNA/LNP are preferentially taken up by myeloid cells irrespective of the tissue.

As part of an IND-enabling multi-dose cynomolgus monkey study, the expression of CAR in immune cell subsets, including CD66abce^+^ granulocytes, CD14^+^ monocytes, CD3^+^ T cells, CD16^+^ NK cells and CD20^+^ B cells was measured in peripheral blood 12 hours after the first intravenous injection. TROP2 CAR expression was determined in circulating immune cells. In agreement with the mouse results, about 25% and 31% of monocytes were CAR-positive at 0.5 mg/kg and 1 mg/kg, respectively. CAR expression in granulocytes was low (**Figure 2B**). Finally, human PBMCs were transfected with TROP2 CAR/LNP *in vitro*. Twenty-four (24) hours after transfection, on average close to 40% of monocytes expressed the CAR (**Figure 2C**). These data demonstrate that the mRNA/LNP used in this study are preferentially taken up by myeloid cells in peripheral blood and are suitable to deliver TROP2 CAR mRNA to myeloid cells.

### Direct anti-tumor effects of TROP2 CAR mRNA/LNP treatment in breast cancer xenograft model

**Figure 3:**
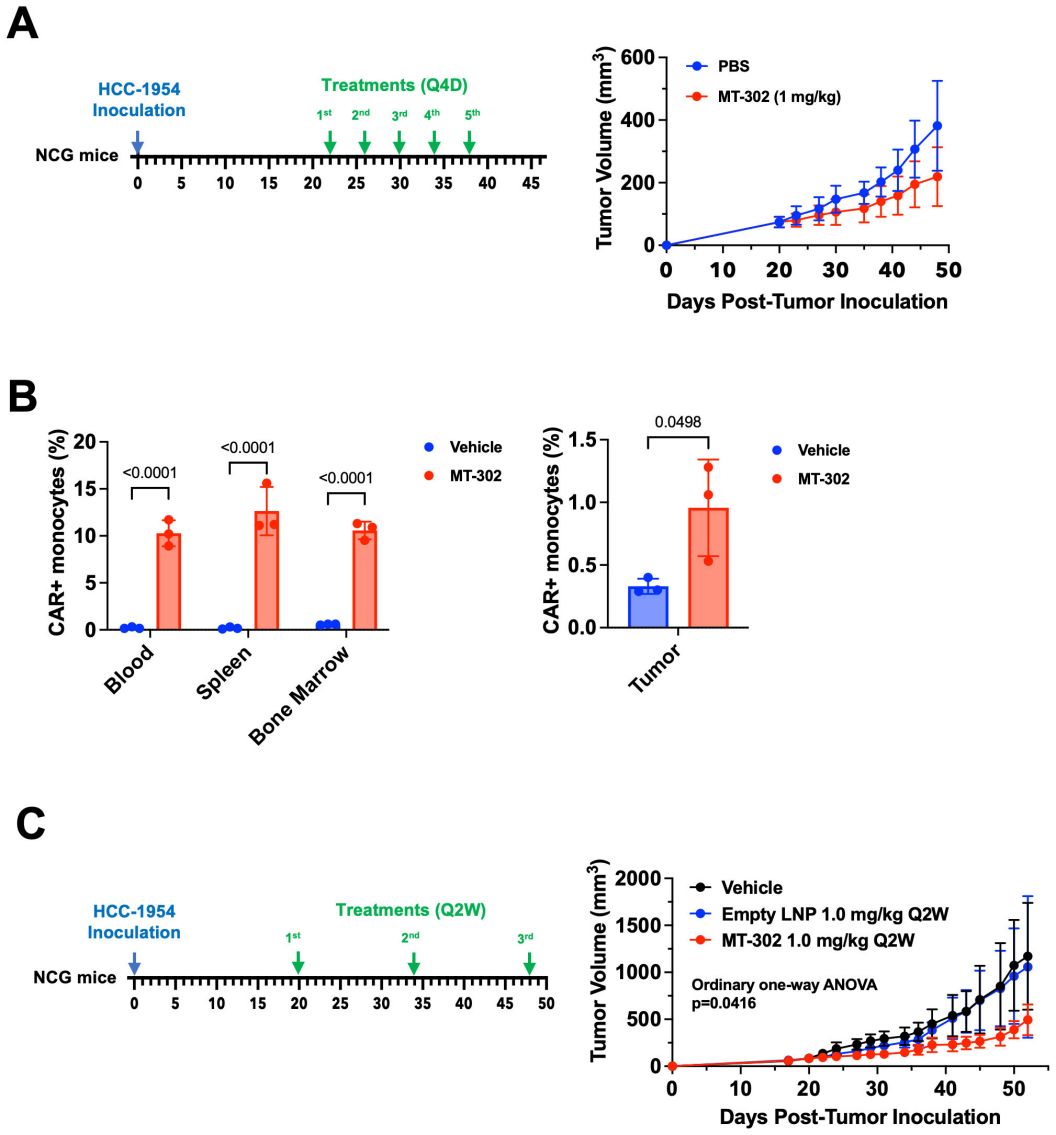
MT-302 shows robust anti-tumor activity in TROP2+ xenograft models. **(A)** Anti-tumor efficacy of MT-302 *in vivo*. NCG mice with established HCC-1954 tumor (TROP2+, s.c.) were treated with MT-302 (1 mg/kg/dose, total 5 doses) or PBS every 4 days (Q4D). Tumor growth was monitored by caliper measurement twice a week. Data shown were average and STD of each group (n= 5 mice per group). **(B)** CAR expression detected in monocytes *in vivo* following MT-302 infusion. NCG mice with established HCC-1954 tumor (tumor volume approximately 500 mm^3^) were dosed i.v. with MT-302 (1 mg/kg) or PBS. Six hours post infusion, blood, spleen, bone marrow and tumor were harvested. Single cell suspensions were prepared from collected tissues and stained for CAR expression. Data shown were average and STD of each group (n= 3 mice per group) for monocytes (CD45+Ly6C+ cells). Statistical analysis was performed by unpaired T-test (two-tailed) for tumor samples, and by Two-Way ANOVA followed by Sidak’s multiple comparisons test for blood, spleen, and bone marrow samples. **(C)** Anti-tumor efficacy of bi-weekly injection of MT-302 *in vivo*. NCG mice with established HCC-1954 tumor (TROP2+, s.c.) were treated with MT-302 (1 mg/kg/dose, total 3 doses) or PBS every 2 weeks (Q2W). Tumor growth was monitored by caliper measurement twice a week. Data shown were average and STD of each group (n= 5 mice per group). Statistical analysis was performed by Ordinary One-Way ANOVA.

To assess the anti-tumor efficacy of TROP2 CAR mRNA/LNP we established an HCC-1954 breast cancer xenograft model in NCG mice. Of note, NCG mice are immunodeficient mice lacking T cells, B cell, and NK cells, but still have an intact myeloid cell compartment. This makes the model suitable for the evaluation of direct cytotoxic effect of TROP2 CAR-expressing myeloid cells. Five (5) doses of TROP2 CAR mRNA/LNP (1 mg/kg) given in 4-day intervals, significantly reduced tumor growth compared to the PBS vehicle control (**Figure 3A**). CAR expression on monocytes was measured 24 hours after the first dose. In line with the observations in non-tumor bearing C57BL/6 mice, approximately 10% to 15% of Ly6C^+^ monocytes in peripheral blood, spleen and bone marrow were CAR-positive (**Figure 3B**). Importantly, TROP2 CAR mRNA/LNP treatment resulted in a significant increase of Ly6C^+^ myeloid cells in the tumor microenvironment. The percentage of CAR-positive cells doubled from 0.5% to about 1% (**Figure 3B**). In a different study with the same established HCC-1954 breast cancer tumors, fewer doses given every 2 weeks still showed significant anti-tumor effect compared to PBS vehicle and empty LNP (without RNA) controls (**Figure 3C**). The treatment was well tolerated with no observed adverse event or loss of body weight (**Supplementary Figure 2**). The results highlight the capacity of TROP2 CAR myeloid cells to migrate to the tumor and exert direct anti-tumor effects.

### A surrogate anti-gp75 CAR LNP shows anti-tumor activity in the syngeneic B16/F10-OVA melanoma model by re-polarizing immunosuppressive tumor microenvironment

The programming of myeloid cells is an attractive therapeutic approach because of their role in the orchestration of a comprehensive immune response. They not only have the potential to kill tumor cells directly through phagocytosis or antibody-mediated cellular cytotoxicity, but also promote other innate and adaptive immune mechanism. To study the broader impact of *in vivo* CAR-programmed myeloid cells, we conducted pharmacological studies in immunocompetent C57BL/6 mice. Due to the lack of cross-reactivity of the hRS7-derived scFv with mouse TROP2, we established a B16/F10-OVA mouse melanoma model that expresses the gp75 (also known as tyrosinase-related protein-1 or TRP-1) antigen. Of note, B16/F10 tumors are refractory to immunotherapy with monotherapy CAR-T cell or checkpoint inhibition (32).

Prior to testing the gp75 CAR mRNA/LNP *in vivo*, we demonstrated that electroporation of monocytes with gp75 CAR mRNA produced a functional CAR. The murine BMDMs expressed gp75 CAR after mRNA electroporation (**Supplementary Figure 3A**) and phagocytosed the gp75^+^ B16/F10-OVA cells *in vitro* (**Supplementary Figure 3B**).

**Figure 4:**
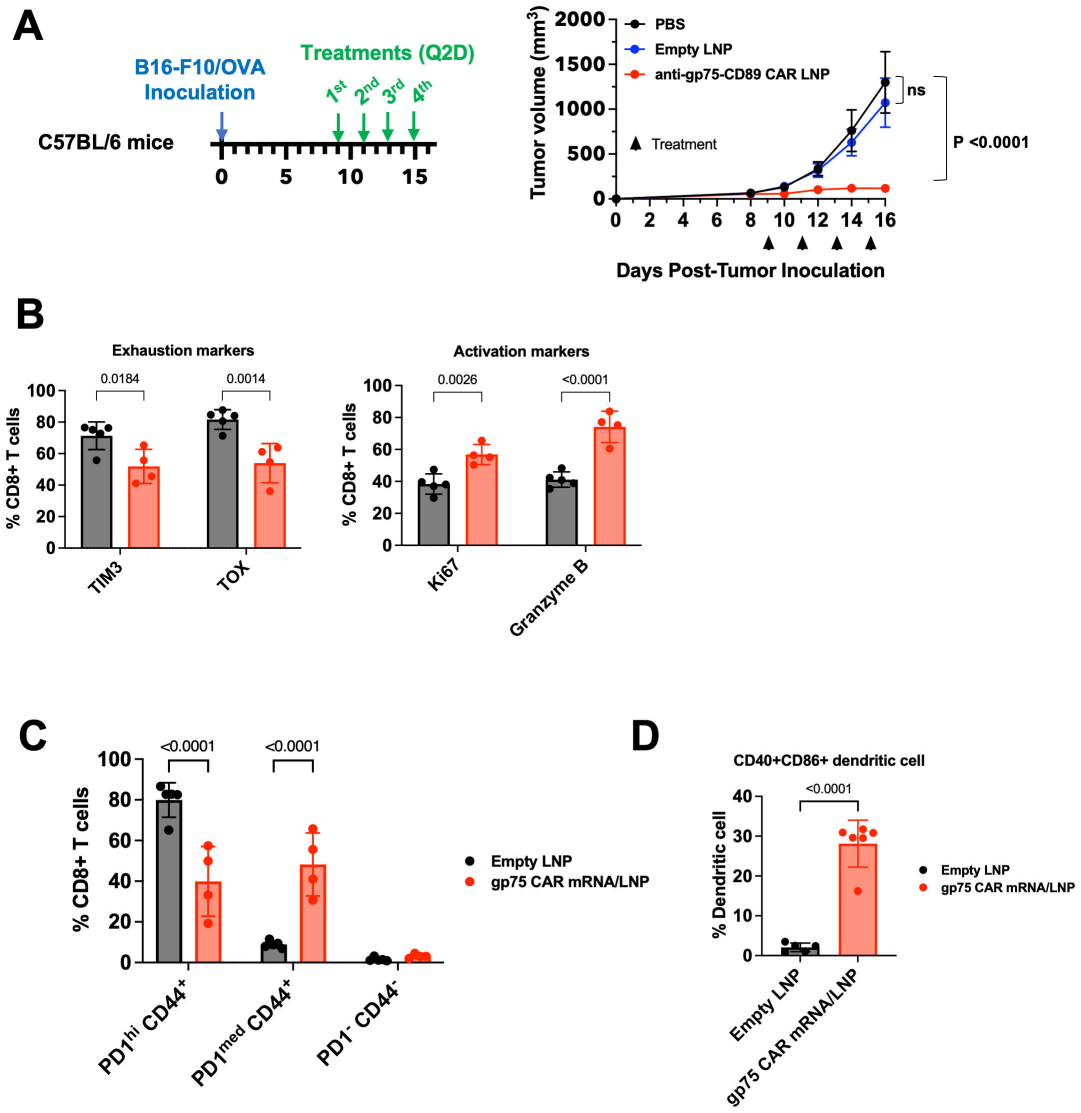
gp75 CAR mRNA/LNP shows anti-tumor activity in the B16/F10 syngeneic melanoma model and remodels the TMEs. **(A)** Anti-tumor efficacy of surrogate gp75 CAR mRNA/LNP in syngeneic mouse melanoma model. C57BL/6 mice were inoculated s.c. with B16/F10-OVA tumor cells on day 0. Upon tumor establishment, PBS, Empty LNP or gp75 CAR mRNA/LNP were injected i.v. at 2 mg/kg/dose every 2 days. Data shown are mean ± SEM (n=6). **(B, C)** Flow cytometry analysis of CD8^+^ T cell phenotypes in tumors 24 h post 4th dose. **(B)** Treatment with gp75 CAR mRNA/LNP significantly increased the frequency of proliferating (CD8^+^ Ki67^+^) and cytolytic (Granzyme B^+^) CD8^+^ T cells, and simultaneously reduced the percentage of TIM3^+^ and TOX^+^ exhausted T cells. Statistical analysis was performed by Two-Way ANOVA followed by Sidak’s multiple comparisons test. **(C)** Treatment with gp75 CAR mRNA/LNP reduces the percentage of memory CD44^+^ CD8^+^ T cells with exhaustion phenotype indicated by high PD1 expression. Statistical analysis was performed by Two-Way ANOVA followed by Sidak’s multiple comparisons test. **(D)** Treatment with gp75 LNP significantly increased activated dendritic cells (CD40^+^ CD86^+^). Statistical analysis was performed using an unpaired t-test (two-tailed).

Once tumors reached a size of 50 mm^3^, mice were treated with 4 doses of 2 mg/kg gp75 CAR mRNA/LNP every two days. Compared to vehicle and empty LNP control groups, treatment with gp75 CAR mRNA/LNP demonstrated robust anti-tumor response (**Figure 4A**). At the end of the study on day 16, tumors were harvested to assess the impact of the treatment on the tumor microenvironment. Tumor infiltrating CD8+ T cells were identified with the gating strategy shown in **Supplementary Figure 4A**. The phenotypes of the tumor CD8+ T cells were analyzed and shown in **Figure 4B** and **Supplementary Figure 4B**. The number of exhausted CD8+ T cells characterized by TIM3 and TOX expression, was reduced in gp75 mRNA/LNP-treated mice. We also observed an increase in the percentage of Ki67+ CD8+ proliferating T cell and upregulation of cytotoxic granules (granzyme B). Likewise, a decrease in the percentage of PD-1^hi^/CD44^+^ T cells and an increase in PD-1^med^/CD44^+^ T cells further supported the establishment of an adaptive immune response (**Figure 4C**; **Supplementary Figure 4C**). No difference in the percentage of naïve CD8^+^ T cells (PD-1^-^/CD44^-^) was observed between untreated and treated animals. Furthermore, the dendritic cell phenotype in the spleen was determined 24 hours after the fourth dose, with the gating strategy shown in **Supplementary Figure 5A**. Upon gp75 mRNA/LNP treatment, close to 30% of CD45^+^/CD11c^+^/MHCII^+^ DCs cells were CD40^+^CD86^+^ double-positive (**Figure 4D**, **Supplementary Figure 5B**). CD40^hi^CD86^hi^ DCs have been shown to have superior ability to activate anti-tumor T cells (33).

In a follow-on study using the B16/F10-OVA model, in addition to the empty LNP control, we included a gp75 CAR mRNA with three stop codons at the 5’ end coding region to block the translation of the mRNA. In accordance with the previous study, we observed tumor growth inhibition after injection of two doses of 1 mg/kg gp75 mRNA/LNP. Like the PBS vehicle control group, the LNP-encapsulated CAR mRNA with the stop codons did not inhibit tumor growth ruling out unspecific RNA mediated effects (**Supplementary Figure 6A**). In addition, an elevated IgG1 response against ovalbumin expressed by B16/F10-OVA tumor cells was detected in animals treated with the gp75 mRNA/LNP formulation. The mRNA construct with the stop codons did not elicit an antibody response (**Supplementary Figure 6B**).

In summary, the findings in immunocompetent tumor models illustrate the broader impact of CAR-programmed myeloid cells on the adaptive anti-tumor response.

## Discussion

The challenges of CAR-T cell therapies in solid cancer highlight the need for novel treatment modalities. Myeloid cells present an attractive cell population for engineering due to their unique properties to readily infiltrate tumors, kill cancer cells via numerous mechanisms and importantly, produce inflammatory cytokines and chemokine that support the establishment of an adaptive immune response. Here, we describe the *in vivo* engineering of monocytes to express a TROP2 specific CAR for the treatment of solid tumors. Unlike traditional CAR-engineered cell therapies, which require isolation and manipulation of the cells outside the body, this novel therapy bypasses the complex *ex vivo* processing by directly programming myeloid cells in patients using LNP-encapsulated CAR mRNA. Several unique features of the therapy are essential for selective expression and activity of the CAR in myeloid cells.

To harness the full potential of myeloid cells, we designed a novel CAR construct that incorporates CD89, a receptor that is natively expressed on myeloid cell and therefore naturally integrated into myeloid cell signaling circuits. Of note, the short cytoplasmic domain of CD89 contains no known signal transduction domains. To mediate IgA-dependent phagocytosis of microbes and cell activation, CD89 requires the association with FcRγ, an ITAM motif containing signaling adapter molecule (34, 35). Further, FcRγ is not only essential for the function, but also expression of Fc receptors (31). Originally designed for application in T cells, traditional CARs harbor the CD3ζ signaling domain, a component of the T cell receptor complex not found in myeloid cells. Nevertheless, similarities between CD3ζ and FcRγ prompted engineering of *in vitro* differentiated macrophages with CD3ζ CARs (29). While CD3ζ can trigger phagocytic activity, it remains unclear whether CD3ζ is fully compatible with myeloid signaling. We provide evidence that the CD89-based TROP2 CAR associates with the FcRγ signaling chain and demonstrate that the TROP2 CAR is functional: (i) Upon TROP2 binding, CAR-positive THP1-Dual^®^ reporter cells upregulate NFκB and interferon signaling, both hallmarks of myeloid cell activity. (ii) Primary human monocytes electroporated with the TROP2 CAR mRNA lysed target-positive SKOV3 tumor cells and (iii) secreted inflammatory cytokines and chemokines. To accomplish appropriate myeloid cell activation by the CAR and meet the requirement for LNP-based *in vivo* delivery, we purposefully created a CAR that separates antigen binding from cell activation. The CAR is only able to activate cells that endogenously provide the FcRγ signal adapter, namely myeloid cells and NK cells. This cell type specific expression of FcRγ combined with the preferred uptake of the LNPs by monocytes, results in CAR activity that is restricted to myeloid cells thereby avoiding off-target activity in other cell types.

Over the past years, several LNP-delivered RNA therapies, including patisiran for the treatment of ATTR amyloidosis (16) and two RNA-based COVID-19 vaccines (15) have been approved in the US. For the first time, we describe the use of LNPs to deliver CAR mRNA to myeloid cells directly *in vivo* as a promising approach to overcome the costly and complex *ex vivo* generation of CAR-engineered cells. In our biodistribution study in non-tumor bearing mice, the TROP2 mRNA/LNP were readily taken up by cells of the myeloid lineage, namely CD11b^+^/Ly6C^+^ monocytes and to a lesser extent by CD11c^+^MHCII^+^ dendritic cells. The percentage of cells expressing the TROP2 CAR was consistent between peripheral blood, spleen and bone marrow. Further, the lack of NFκB activation in FcRγ-negative Jurkat-Dual™ cells suggests that the TROP2 CAR does not activate cells that lack FcRγ, such as lymphocytes or non-immune cells. This cell type-specific activity is critical for an improved safety profile of intravenously administered CAR mRNA/LNP. Consistent with the expression profile in mouse PBMCs, a similar dose dependency and percentage of CAR-expressing CD14^+^ monocytes were observed in cynomolgus monkeys after a single infusion of TROP2 CAR/LNP. Likewise, when human PBMCs were transfected with TROP2 CAR/LNP, the same CAR expression profile was detected. Collectively, across species the results demonstrate that the non-targeted LNP formulation used in this study is suitable for delivery of CAR mRNA to a high percentage of myeloid cells.

Recently, CAR-T cell therapies targeting TROP2 have been described (36, 37). While there has been some success in early clinical trials in treating patients with neurological cancers (38, 39), mesothelioma (40, 41), and other tumor types (42), treatment of solid tumors with CAR-T cells has proven to be difficult. Multiple factors have been described to limit the benefit of CAR-T cells therapies in solid cancers, including the lack of CAR-T persistence and trafficking to the tumor as well as immunosuppressive mechanisms in the TME. Therefore, many of the new CAR-T cell therapies in preclinical development incorporate enhancements. Among them are (i) the secretion of cytokines such as IL-7, IL-12, IL-15 or IL-18, to increase cell persistence and T cell cytotoxicity, (ii) armoring the cells against TGFβ-mediated inhibitory signals in the hostile TME or (iii) the production of chemokines such as CCL19 or CXCR2 to improve T cells infiltration to the tumor (43, 44). Despite promising preclinical results, after systemic administration of CCL19 and IL-7 armored CAR-T cells, stable disease was the best objective response in a phase 1 clinical trial. These results underscore that armored CAR-T cells may still be limited by inhibitory factors in human tumors (45). Myeloid cells have unique characteristics that are different form T cells and thus, provide an alternative or complementary approach to CAR-T cells. Unlike T cells, inflammatory blood monocytes have a high capacity to traffic to tissues. This property is mediated by the interaction of the chemokine receptor CCR2 with CCL2, a chemokine secreted by infected tissues or tumors. For myeloid cells to maintain their inflammatory phenotype in the TME, we harnessed CD89, a receptor that naturally triggers an IgA-mediated inflammatory pathway (20). Once in the tumor tissue, myeloid cells equipped with CARs can shape the tumor microenvironment in different ways. They can kill tumor cells via phagocytosis or TNFα-mediated mechanism or through the secretion of pro-inflammatory cytokines and chemokines can recruit other immune cells thereby establishing a broader adaptive immune response. While more work is required to fully elucidate the mechanism of CAR myeloid cells in the tumor, the data presented here emphasize the role of inflammatory myeloid cells for cancer therapy. Treatment of mice with CAR mRNA/LNP resulted in robust anti-tumor activity in xenograft and syngeneic mouse models. In an HCC-1954 subcutaneous breast cancer xenograft mouse model, intravenous delivery of TROP2 mRNA/LNP significantly inhibited tumor growth. Of relevance, anti-tumor activity correlated with the infiltration of CAR^+^ myeloid cells into the tumors. Due to the lack of a lymphocyte compartment, the observed anti-tumor efficacy in NCG mice is limited to myeloid cells, thereby underestimating the full anti-tumor potential of CAR myeloid cels. Effects of CAR myeloid cells on the adaptive immune system are absent in xenograft models. Therefore, we developed a gp75 CAR surrogate therapy that enabled us to study the impact of CAR myeloid cells on the broader immune system in C57BL/6 immunocompetent mice in the context of B16/F10 melanoma. The reduction in T cells exhaustion, upregulation of granzyme B, and increased proliferation of cytotoxic T cells clearly showed the stimulatory effect on the T cell compartment. Generally, syngeneic mouse tumors such as MC38 or B16/F10 are resistant or respond very poorly to CAR-T cell therapy. Only pre-conditioning with radiation (46) or in the case of B16/F10, pretreating the tumor with an anti-TRP1 antibody in combination with further CAR-T cell enhancements, makes these tumors responsive to CAR-T cells therapy (47). Of note, treatment of established B16/F10-OVA tumors with gp75 CAR mRNA/LNP significantly reduced tumor growth without preconditioning or combination therapy. These results suggest that CAR engineered myeloid cells through their polyfunctional effects on the innate and adaptive immune response provide anti-tumor functions that CAR-T cells lack.

Our work together with that of others demonstrates the benefits of engineering myeloid cells to express inflammatory cytokines or CARs (10, 29, 48, 49). *In vivo* engineering of myeloid cells by intravenous infusion of CAR mRNA/LNP product bypasses the complex *ex vivo* manipulation of cells and combined with no requirement for lymphodepleting chemotherapy conditioning, holds great promise for future cancer therapies. Encouraged by the preclinical data, the TROP2 CAR mRNA/LNP drug development candidate, dubbed MT-302, is being tested in patients with advanced or metastatic epithelial tumors (NCT05969041).

## Data Availability

The raw data supporting the conclusions of this article will be made available by the authors, without undue reservation.
