# Supplementary material for: In vivo programmed myeloid cells expressing novel chimeric antigen receptors show potent anti-tumor activity in preclinical solid tumor models
Source: Front Immunol. 2024 Dec 13;15:1501365. doi: 10.3389/fimmu.2024.1501365 (PMC11671302; doi:10.3389/fimmu.2024.1501365)
Supplement: Supplementary file 1 [file DataSheet1.docx]

**In vivo Programmed Myeloid Cells Expressing Novel Chimeric Antigen Receptors Show Potent Anti-tumor Activity in Preclinical Solid Tumor Models**

Shannon Argueta^1^, Yuxiao Wang^1^, Hongyun Zhao, Neha Diwanji, Michael Gorgievski, Edward Cochran, Ewa Grudzien-Nogalska, Josephine D'Alessandro, Bruce McCreedy, Thomas Prod’homme, Robert Hofmeister*, Jian Ding and Daniel Getts*

^1^These authors contributed equally to this work

*Correspondence Authors

Myeloid Therapeutics, Inc., 300 Technology Square, Cambridge, MA 02139

**
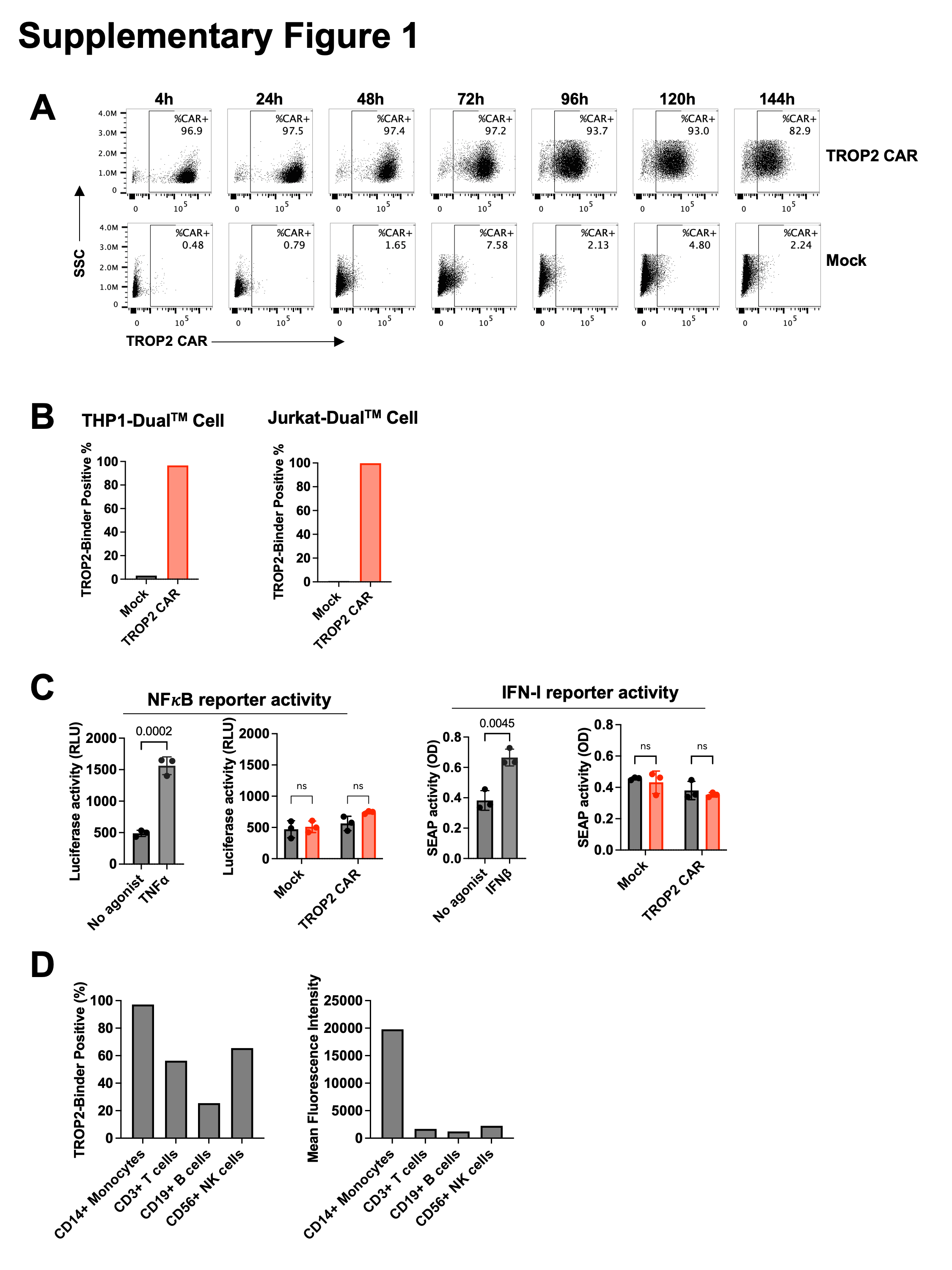
**

**Supplementary Figure 1. Expression of TROP2 CAR is influenced by the presence of FcR𝛾 chain. (A)** Flow cytometry dot plots showing expression levels of TROP2 CAR over 7 days post electroporation, related to Figure 1B. (**B**) Expression levels of TROP2 CAR in THP1-Dual and Jurkat-Dual reporter cells 24 h post electroporation. (C) TROP2 CAR did not induce NF-𝜅B and IFN-I pathways activation in Jurkat-Dual^TM^ reporter cell which lacks FcR𝛾. Stimulation with agonist cytokines including TNF𝛼 or IFNβ are shown as positive control. . Statistical analysis was performed by Two-Way ANOVA followed by Sidak’s multiple comparisons test for comparing unstimulated and TROP2-stimulated conditions, and an unpaired t-test (two-tailed) for comparing no agonist vs agonist cytokine stimulated conditions. **(D)** Expression levels of TROP2 CAR in different immune cell subsets in PBMC 24h post electroporation.

**
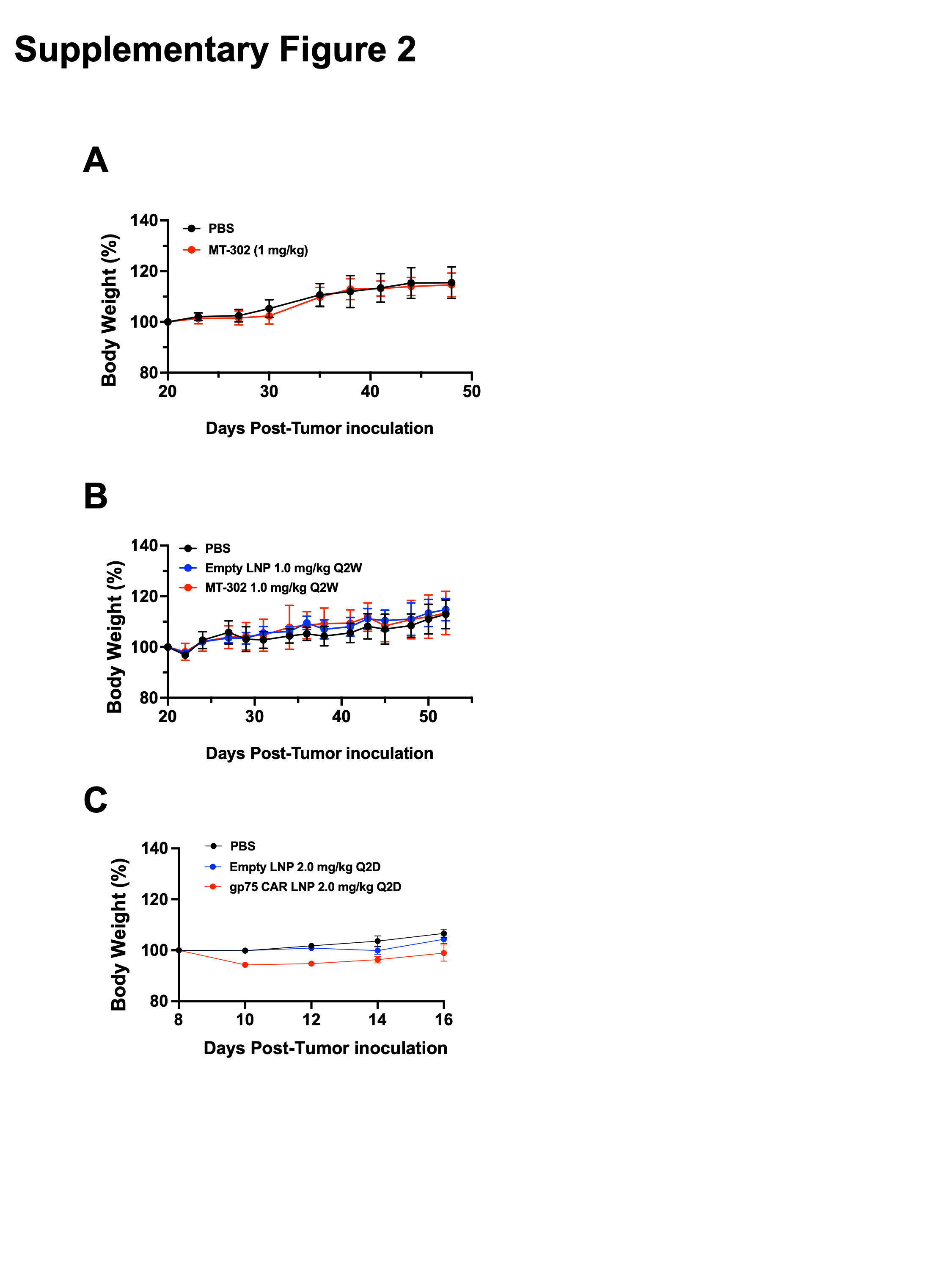
**

**Supplementary Figure 2. Body weight of tumor bearing mice post treatment**

NCG mice bearing HCC-1954 tumors were treated with vehicle, empty LNP or MT-302 as shown in the Figure 3A and Figure 3C. The body weight change (as percentage to the baseline bodyweight right before treatments) were shown for animals treated with MT-302 every 4 days (A) or every week (B). Data shown were average and STD of each group (n= 5 mice per group). (C) C57BL/6 mice bearing B16/F10-OVA tumors were treated with PBS, empty LNP or gp75-CAR LNP as shown in Figure 4A. Body weight change (as percentage to the baseline bodyweight right before treatments at day 8) were shown.


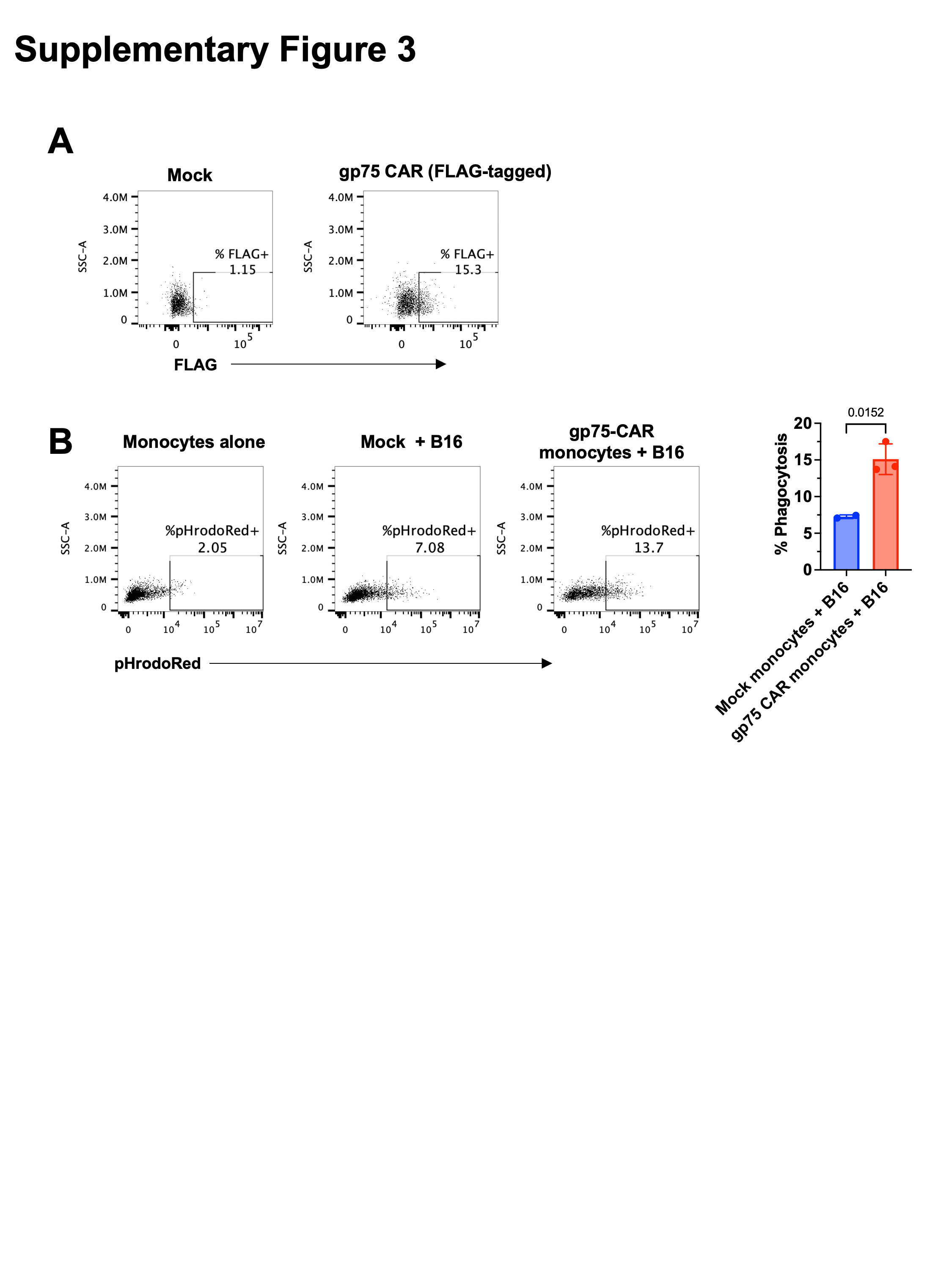


**Supplementary Figure 3. Murine bone marrow derived gp75 CAR monocytes phagocytoses B16/F10-OVA melanoma cells and secrete cytokines and chemokines.** **(A)** Expression levels of FLAG-tagged gp75 CAR 24h after transfection into murine bone marrow monocytes using Lonza 4D nucleofector. **(B)** gp75 transfected monocytes showed increased phagocytosis against B16/F10-OVA melanoma cells. **(C)** incubation of gp75 CAR monocytes with B16/F10-OVA tumor cells resulted in secretion of pro-inflammatory cytokines and chemokines.


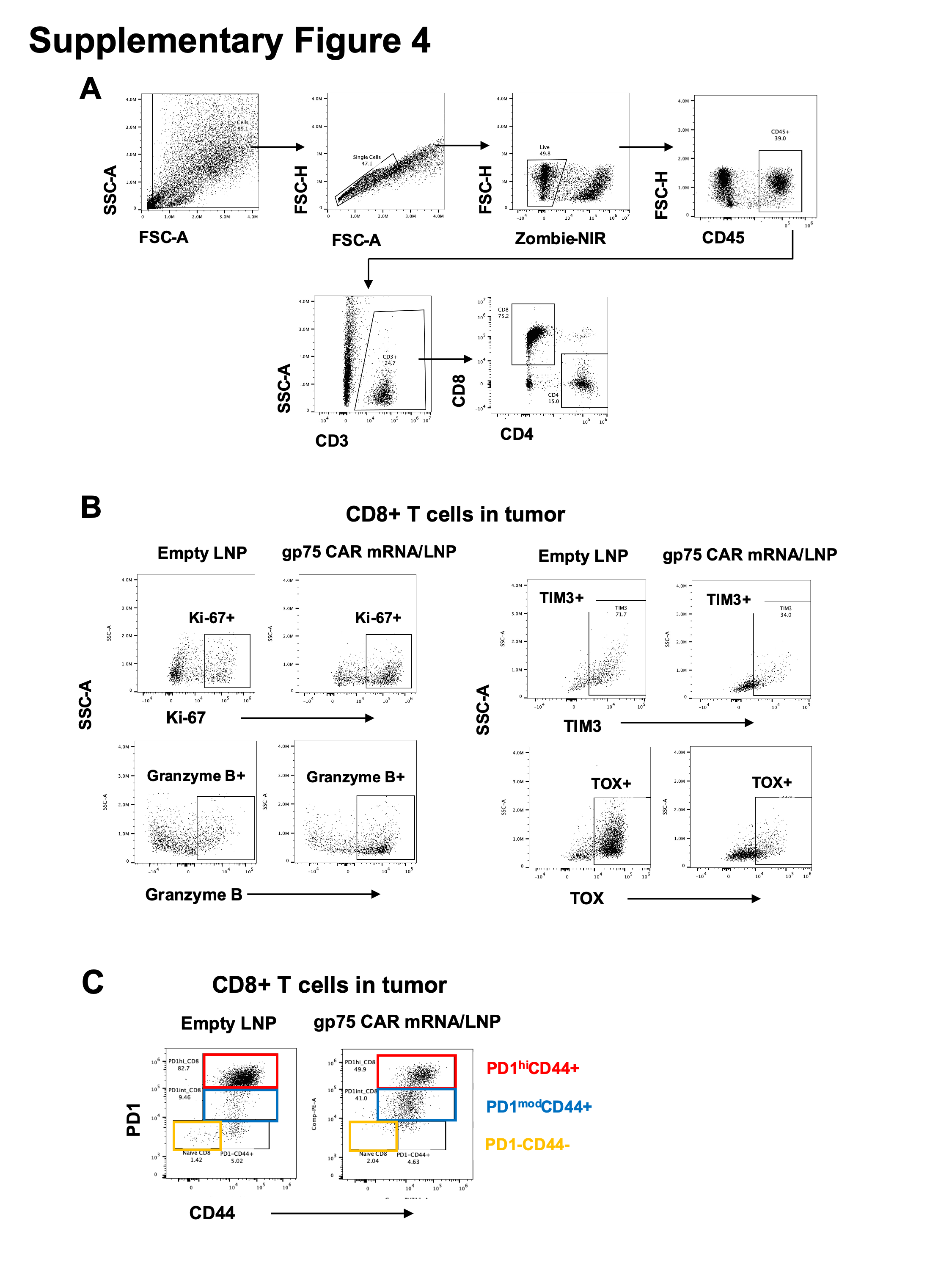


**Supplementary Figure 4. Gating strategy to identify CD8+ T cells in tumor and representative flow plots showing gp75 CAR delivery modifies tumor CD8+ T cells**

(**A**) Gating strategy to identify CD8+ T cells in tumor. Single cell suspensions were obtained from harvested tumors and were analyzed by flow cytometry. In brief, the steps shown in (A) were followed to exclude debris, doublets, dead cells and CD45- cells, sequentially. Among the gated live, single, CD45+ cells, T cells were identified as CD3+ cells. Among CD3+ T cells, CD8+ T cells were identified as CD8+CD4- population. (**B**) Increased level of Ki-67+ and Granzyme B+ CD8+ T cells as well as decreased levels of TIM3+ and TOX+ CD8+ T cells were observed for gp75 CAR LNP treated mice than for empty LNP treated mice. Representative results of 4 mice (gp75 CAR LNP treated) or 5 mice (empty LNP treated) are shown. (**C**) gp75 CAR LNP treatment decreased levels of exhausted memory CD8+ T cells (PD1^hi^CD44+) and increased levels of activated memory CD8+ T cells (PD1^mod^CD44+). Representative results of 4 mice (gp75 CAR LNP treated) or 5 mice (empty LNP treated) are shown.


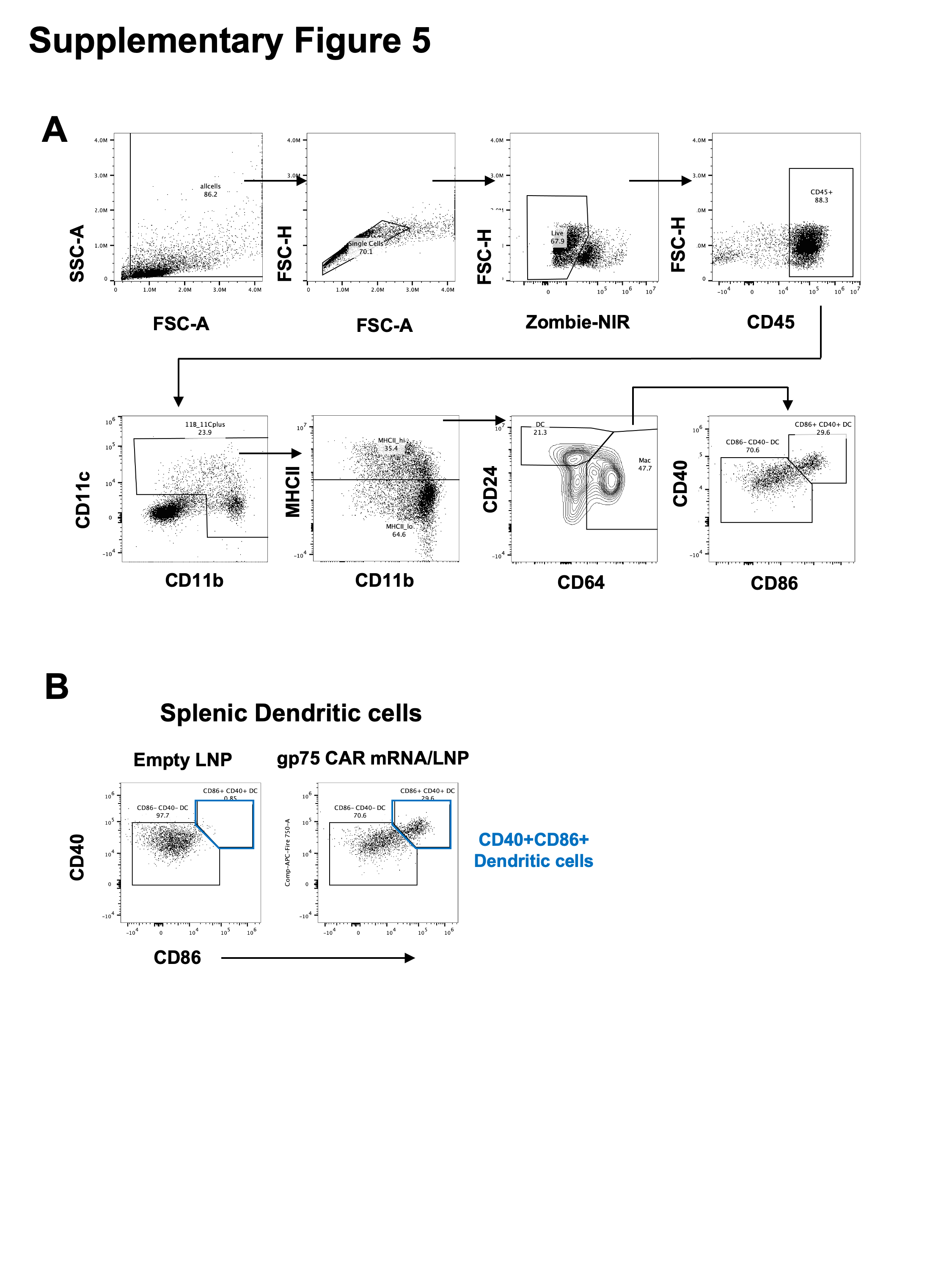


**Supplementary Figure 5. Gating strategy to identify DCs in spleen and representative flow plots showing gp75 CAR delivery promotes DC activation**

(**A**) Gating strategy to identify DCs in spleen. Single cell suspensions were obtained from harvested spleens and were analyzed by flow cytometry. In brief, the steps shown in (A) were followed to exclude debris, doublets, dead cells and CD45- cells, sequentially. Among the gated live, single, CD45+ cells, DCs were identified as CD11c^+^MHC-II^+^CD64^+^CD24^mod^ cells. CD86 and CD40 expression levels were then evaluated for DCs activation status (**B**) Increased level of CD40+CD86+ activated DCs were observed for gp75 CAR LNP treated mice than for empty LNP treated mice. Representative results of 6 mice from each treatment group are shown.


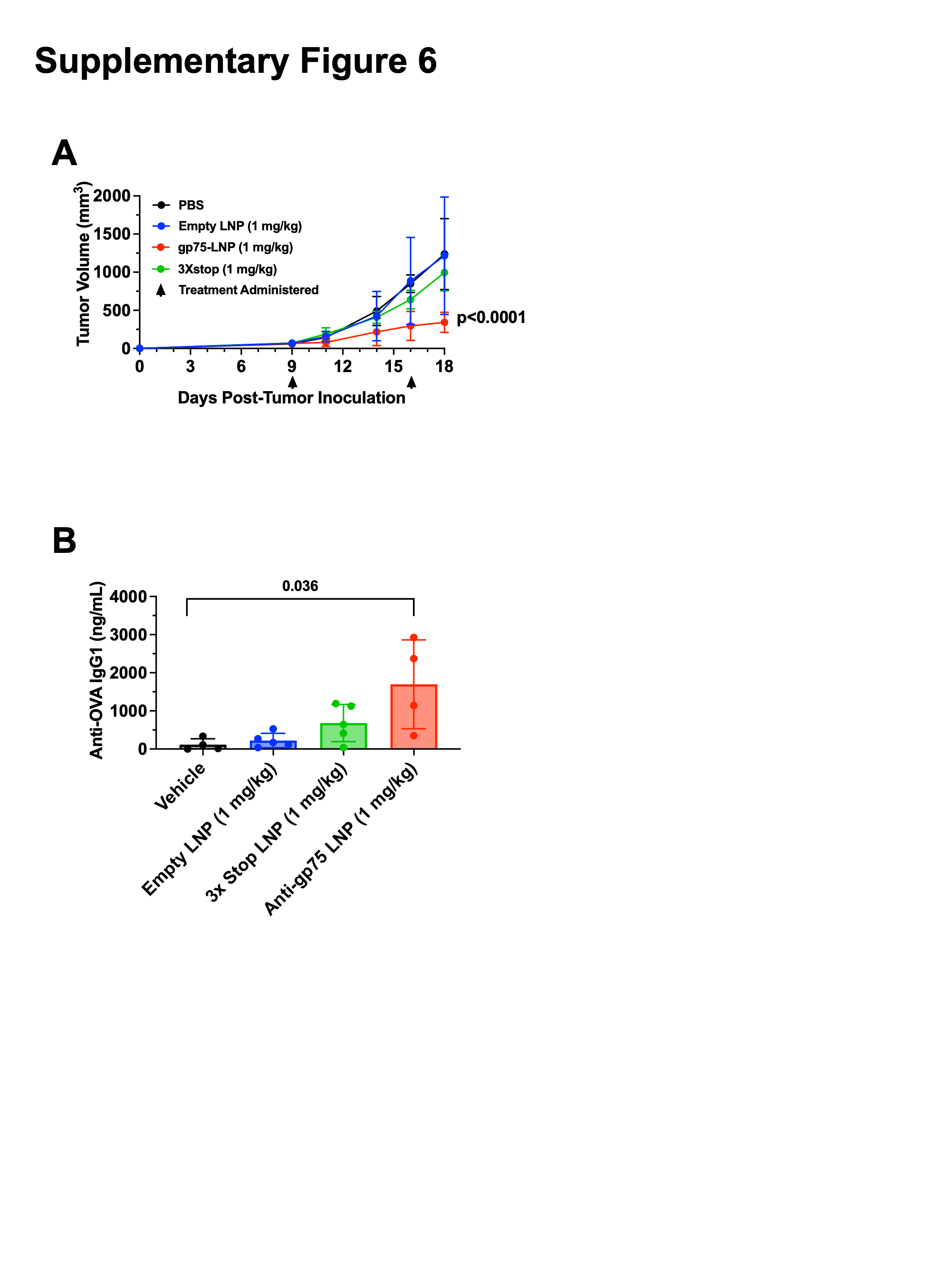


**Supplementary Figure 6. gp75 CAR delivery to myeloid cells boosts humoral immune response in syngeneic model.**

(**A**) Anti-tumor efficacy of surrogate gp75 CAR mRNA/LNP in syngeneic mouse melanoma model. C57BL/6 mice were inoculated s.c. with B16-F10/OVA tumor cells on day 0. Upon tumor establishment (day 9), PBS, Empty LNP, gp75 CAR mRNA with 3 copies of stop codon in LNP (3Xstop) or gp75 CAR mRNA/LNP were injected i.v. at 1 mg/kg/dose once a week. Tumor growth was monitored by caliper measurement twice a week. Data shown were average and STD of each group (n= 4 mice per group). (**B**) Elevation of anti-OVA Ab response induced by gp75 CAR mRNA/LNP. On day 18 after tumor inoculation, 48 hours after 2^nd^ LNP infusion, serum were collected from all treatment groups. Anti-OVA IgG1 level in serum was quantitated by ELISA. Data shown were average and STD of each group (n= 4 mice per group).
